# Stereotactic body radiotherapy for centrally located early-stage non-small cell lung cancer or lung metastases from the RSSearch^®^ patient registry

**DOI:** 10.1186/s13014-015-0417-5

**Published:** 2015-05-15

**Authors:** Joanne N. Davis, Clinton Medbery, Sanjeev Sharma, John Pablo, Frank Kimsey, David Perry, Alexander Muacevic, Anand Mahadevan

**Affiliations:** The Radiosurgery Society®, 1350 Dell, Suite 105, Campbell, CA 95008 USA; Department of Radiation Oncology, St. Anthony Hospital, Oklahoma City, OK USA; Department of Radiation Oncology, St. Mary’s Medical Center, Huntington, WV USA; Department of Radiation Oncology, St. Joseph/Candler Hospital, Savannah, GA USA; Department of Radiation Oncology, Erlanger Health System, Chattanooga, TN USA; Department of Radiation Oncology, Medstar Franklin Square Medical Center, Baltimore, MD USA; European CyberKnife Center Munich-Grosshadern and Munich University, Munich, Germany; Department of Radiation Oncology, Beth Israel Deaconess Medical Center, Harvard Medical School, Boston, MA USA

**Keywords:** Stereotactic body radiotherapy, Lung cancer, Central lung tumors, Lung metastasis, Patient registry, RSSearch®

## Abstract

**Background:**

The purpose of this study was to evaluate treatment patterns and outcomes of stereotactic body radiotherapy (SBRT) for centrally located primary non-small cell lung cancer (NSCLC) or lung metastases from the RSSearch^®^ Patient Registry, an international, multi-center patient registry dedicated to radiosurgery and SBRT.

**Methods:**

Eligible patients included those with centrally located lung tumors clinically staged T1-T2 N0, M0, biopsy-confirmed NSCLC or lung metastases treated with SBRT between November 2004 and January 2014. Descriptive analysis was used to report patient demographics and treatment patterns. Overall survival (OS) and local control (LC) were determined using Kaplan-Meier method. Toxicity was reported using the Common Terminology Criteria for Adverse Events version 3.0.

**Results:**

In total, 111 patients with 114 centrally located lung tumors (48 T1-T2,N0,M0 NSCLC and 66 lung metastases) were treated with SBRT at 19 academic and community-based radiotherapy centers in the US and Germany. Median follow-up was 17 months (range, 1–72). Median age was 74 years for primary NSCLC patients and 65 years for lung metastases patients (p < 0.001). SBRT dose varied from 16 – 60 Gy (median 48 Gy) delivered in 1–5 fractions (median 4 fractions). Median dose to centrally located primary NSCLC was 48 Gy compared to 37.5 Gy for lung metastases (p = 0.0001) and median BED_10_ was 105.6 Gy for primary NSCLC and 93.6 Gy for lung metastases (p = 0.0005). Two-year OS for T1N0M0 and T2N0M0 NSCLC was 79 and 32.1 %, respectively (p = 0.009) and 2-year OS for lung metastases was 49.6 %. Two-year LC was 76.4 and 69.8 % for primary NSCLC and lung metastases, respectively. Toxicity was low with no Grade 3 or higher acute or late toxicities.

**Conclusion:**

Overall, patients with centrally located primary NSCLC were older and received higher doses of SBRT than those with lung metastases. Despite these differences, LC and OS was favorable for patients with central lung tumors treated with SBRT. Reported toxicity was low, although low grade toxicities were observed in patients where dose tolerances approached or exceeded published guidelines. Prospective studies are needed to further define the optimal SBRT dose for this cohort of patients.

**Trial registration:**

Clinicaltrials.gov Identifier: NCT01885299

## Background

Stereotactic body radiotherapy (SBRT) is a treatment option for Stage I patients who are medically inoperable or who refuse surgery. SBRT has achieved LC and OS rates comparable to lobectomy in non-randomized studies in medically inoperable or elderly patients [1–5]. SBRT can also achieve high LC and low toxicity in patients with peripheral lung metastases and limited oligometastatic disease [6–8]. There is increasing interest in SBRT for the treatment of centrally located lung lesions and identifying an optimal efficacious SBRT dose that is safe to deliver. Early reports of SBRT for the treatment of centrally located lung tumors indicated high rates of treatment-related toxicities and treatment-related deaths [9, 10]. In a Phase II study, Timmerman *et al.* reported excessive Grade 3–5 toxicity in patients with centrally located NSCLC treated with 60 – 66 Gy delivered in 3 fractions [10]. Two-year freedom from severe toxicity was only 54 % in patients with central tumors compared to 83 % for patients with peripheral tumors. Song *et al.* reported 33 % Grade 3–5 toxicity in patients with centrally located Stage I NSCLC treated with 40 – 60 Gy in 3–4 fractions [9]. Eight of nine patients with tumors adjacent to the bronchus resulted in bronchial strictures and secondary loss of lung volume. The close proximity of normal critical organs including the heart, bronchus, esophagus and trachea to centrally located lung tumors, create a challenge of achieving an efficacious SBRT dose while limiting dose to normal tissues.

In more recent studies, SBRT treatment of centrally located lung tumors has shown promising results in a subset of patients. In these studies, SBRT doses of 23 – 70 Gy delivered over 1–10 fractions to central lung tumors resulted in LC rates of 60 – 100 % and OS rates of 12 – 74 % at 2–3 years [11–19]. Acceptable toxicity profiles were achieved when strict normal tissue dose constraints were adhered to, [11, 14, 17] however, some authors report Grade 3 and higher pulmonary toxicities with some Grade 5 toxicity related to the SBRT treatment [12, 13, 16, 18, 19]. The Radiation Therapy Oncology Group (RTOG) 0813 study was initiated to assess five-fraction SBRT regimens for early-stage, centrally located NSCLC in medically inoperable patients. The results of this study, however, will not be available for several more years. In the meantime, community-based practices and academic centers are treating patients with centrally located lung tumors with large variations in SBRT dose and fractionation regimens [20]. Until the optimal SBRT dose/fractionation regimens and doses to normal tissues for central tumors are determined, it will be critical for treating centers to combine data collection efforts to gain clinical evidence to support or redefine SBRT for centrally located tumors.

The RSSearch® Patient Registry is an international database designed to standardize the collection of screening, treatment and outcome data for patients treated with radiosurgery and SBRT [21]. RSSearch® currently includes over 15,000 enrolled patients treated with radiosurgery/SBRT. Initial analysis of RSSearch® reported lung tumors as the most common extra-cranial treatment location, with over 5,500 patients with thoracic lesions treated with SBRT [21]. The purpose of this study is to report on treatment management practices, toxicity, OS and LC of centrally located early-stage NSCLC and lung metastases from patients enrolled in RSSearch® Patient Registry, thus representing a real-world setting. Patients with recurrent lung lesions or prior radiation therapy in the SBRT-treated area were excluded from the analysis.

## Methods

A retrospective analysis of patients with centrally located, early stage T1-T2N0M0 NSCLC or lung metastases and enrolled in the RSSearch® Patient Registry (Clinicaltrials.gov Identifier: NCT01885299) was performed. The RSSearch® Patient Registry is managed by the Radiosurgery Society®, a non-profit professional medical society. A description of the methodology, database design and initial patient and treatment characteristics of patients enrolled in RSSearch® has been previously reported [21]. The database is housed by an independent third-party, Advertek^SM^, Inc. (Louisville, KY) and meets all requirements to comply with the Health Insurance Portability and Accountability Act (HIPAA) and Safe Harbor Policy to maintain system security, transmission of data and patient confidentiality. All centers treating patients with SBRT clinically are offered and encouraged to participate in RSSearch®. Participation is voluntary and no compensation is provided either to patients or participating centers. Each principal investigator is provided a copy of the RSSearch® Registry protocol, case report forms, sample patient informed consent, and web-based training for data entry and database navigation. Local Institutional Review Board/Ethics Committee (IRB/EC) approval is required at all participating centers. Informed consent was obtained from all patients, as required by individual IRB/ECs, prior to the patient’s data entered into the RSSearch® Registry. The selection of centers for this study included RSSearch® participating centers that treated patients with centrally located T1-T2,N0,M0 NSCLC or lung metastasis with SBRT between November 2004 to January 2014, with complete data entry fields for screening, treatment and follow-up (minimum survival data) for their respective patients. Central lesions were defined as lesions within 2 cm of the bronchial tree, trachea, major vessels, esophagus, heart, pericardium, or brachial plexus. Patients with recurrent lung lesions or prior radiation therapy in the SBRT-treated area were excluded from the analysis. Patients were treated at 18 institutions located in the US and one center located in Munich, Germany.

Because this is a registry, there were no pre-defined treatment planning criteria and treatment planning was done per institutional guidelines. All patients were simulated supine, in the treatment position. Planning computed tomography (CT) scans were obtained above and below the region of interest in expiration, inspiration and free breathing. One mm slice thickness reconstructions in the axial plane were transferred to the treatment planning station. Diagnostic positron emission tomography (PET) scan images were routinely used for image fusion to aid target volume delineation. Target volumes were delineated by the treating physician (radiation oncologist, pulmonologist, or surgeon) using all available imaging studies, typically including at least CT and PET scanning. The gross tumor volume (GTV) was generally used as the clinical target volume (CTV), and a margin of 3–10 mm was used to delineate the planning target volume (PTV). All planning was performed using inverse planning on the MultiPlan® System (Accuray Incorporated, Sunnyvale, CA) allowing non-isocentric, and non-coplanar radiation delivery using either a ray tracing algorithm or Monte Carlo calculations. All patients were treated using CyberKnife® Stereotactic Radiosurgery System (Accuray Incorporated, Sunnyvale, CA). Real time tumor tracking was accomplished using Synchrony® Respiratory Motion Tracking System, which synchronizes the beam delivery with the motion of the target resulting from respiration, without the need to interrupt the treatment or move the patient. All patients were treated according to the respective institutional guidelines. To compare the effects of various treatment protocols with different treatment fraction sizes and doses, the biologically effective dose (BED) was calculated using the linear quadratic model; as BED = D_*_(1 + *d*/α/β) where *D* is the total dose, *d* is the dose per fraction and the α/β ratio for the tumor was 10 Gy. Normal tissue dose restraints were reported by the treating institutions and captured in RSSearch® as the maximum point dose and interquartile range for each structure.

Patient follow-up was performed per institutional guidelines. All participating centers reported follow-up clinical and imaging data. Local control was evaluated independently for each lesion at the participating institution following a modified RECIST (Response Evaluation and Criteria in Solid Tumors) criteria. Local progression was defined as at least a 20 % increase in the size of lesions and/or appearance of one or more lesions in target treatment location, LC defined as disappearance of, decrease in, or no increase in size of the treated lesions. An independent audit by a contracted third party was conducted on 10 % of patients to assess accuracy and completeness of RSSearch® data. De-identified medical records, including consultation reports, diagnostic imaging reports, pathology reports, treatment planning and delivery records, follow-up consultation reports and imaging reports were reviewed and compared to data recorded in RSSearch®. Any missing information or deviations were immediately reported to the participating center and corrections were made prior to data analysis.

Analyses of LC, and OS were calculated using the Kaplan-Meier method. LC was analyzed for each treated tumor whereas analysis of OS was calculated for every patient. Specific cause of death was not reported for all patients in RSSearch® and therefore not evaluated in this study. Subgroups were compared using *X*^*2*^ and log-rank statistics. Values of p < 0.05 were considered statistically significant. For primary central lung tumors significant factors including age, T Stage and BED, were included in a multivariate Cox regression model. Statistical calculations were conducted using GraphPad Prism (La Jolla, CA) and STATA (StatCorp LP, TX). Toxicity was scored according to the National Cancer Institute Common Terminology Criteria for Adverse Events, version 3 (CTCAEv3).

## Results

### Patient and treatment characteristics

From November 2005 to January 2014, 111 patients with centrally located early-stage T1-T2N0M0 NSCLC (47 patients) or lung metastases (64 patients) were treated with SBRT. Table 1 summarizes the patient and tumor characteristics. Patients with centrally located primary NSCLC were significantly older than those with lung metastases (74 years vs 65 years, respectively, p < 0.001). The median Karnofsky Performance Score (KPS) was 80 % (range 60–100) in both cohorts. Sixty-four percent of primary NSCLC patients were considered to be surgically/medically inoperable and 37 % of patients with lung metastases; the remaining patients refused other treatment options. The majority of patients with primary NSCLC (77 %) had no prior treatment, whereas the majority of patients with lung metastases had received prior treatments including chemotherapy (68 %), surgery (26 %), immunotherapy (9 %) and radiofrequency ablation (4 %). Patients that received previous radiation therapy in the SBRT-treated area were excluded from this study. Histological confirmation was available for all primary NSCLC cases, of which 46 % were squamous cell carcinoma, 48 % NSCLC not otherwise specified, and 6 % adenocarcinoma. Fifty-six percent were staged T1N0M0 and 44 % staged T2N0M0. The most common primary sites for lung metastases were colorectal (30 %), kidney (15 %), lung (11 %), breast (11 %) and gynecological (11 %) cancer. With respect to the entire cohort, 46 % of lesions were located near the hilum, two lesions abutted the aorta, three lesions were adjacent to the trachea, and two lesions were adjacent to the mediastinum. The median lesion volume for all lesions was 22 cc (range 0.3 – 280 cc; Table 1), the median lesion volume of primary NSCLC was 24.4 cc (range 2 – 280 cc) and the median lesion volume of lung metastases was 20.6 cc (range 0.3 – 147 cc; Table 1).

**Table 1:** Patient and tumor characteristics

	Primary NSCLC N (%)	Lung Metastases N (%)	p-value
Number of Patients	47	64	
Age (years)			< 0.001
Median	74	65	
Range	(41 – 93)	(35 – 84)	
Gender			0.258
Male	28 (60 %)	31 (48 %)	
Female	19 (40 %)	33 (52 %)	
KPS (%)			0.678
Median	80	80	
Range	60 – 100	60 – 100	
Prior Treatments			
None	36 (77 %)	17 (30 %)	
Chemotherapy	8 (17 %)	39 (68 %)	
Surgery	3 (6 %)	15 (26 %)	
Immunotherapy	0 (0 %)	5 (9 %)	
Radiofrequency Ablation	0 (0 %)	2 (4 %)	
Other	2 (4 %)	3 (5 %)	
Tumor Volume (cc)			0.117
Median	24.4	20.6	
Range	(2 – 280)	(0.3 – 147)	
TNM status (n = 48)			
T1N0M0	27 (56 %)		
T2N0M0	21 (44 %)		
Tumor Histology (n = 48)			
Adenocarcinoma	3 (6 %)		
Squamous Cell Carcinoma	22 (46 %)		
NSCLC not specified	23 (48 %)		

**Fig. 1:**
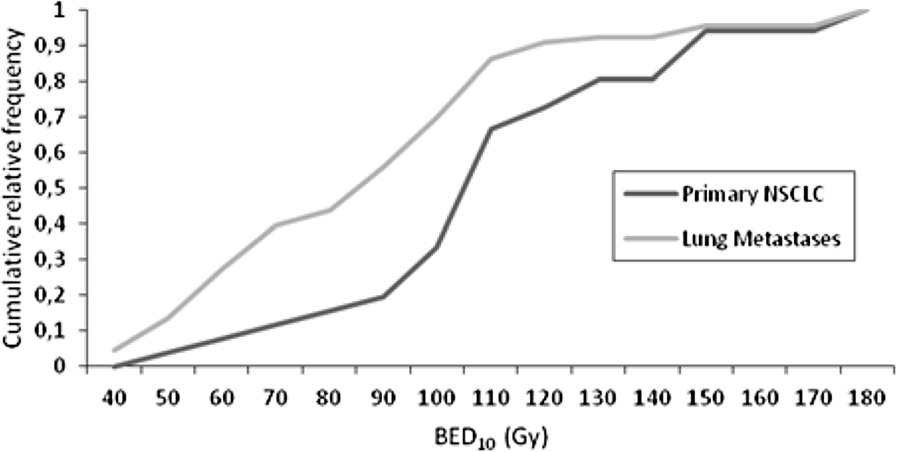
Frequency distribution of BED_10_ for centrally located primary NSCLC (*black*) and lung metastases (*grey*)

SBRT treatment dose was 16 – 60 Gy (median 48 Gy) delivered in 1–5 fractions (median 4 fractions). The median prescribed dose to centrally located primary NSCLC was 48 Gy (range 20 – 60 Gy) compared to 37.5 Gy (range 16 – 60) for lung metastases (p = 0.0001; Table 2), and the median number of fractions delivered to primary NSCLC and lung metastases was 4 and 3, respectively (p = 0.013). Due to the wide variation in dose/fractionation regimens, BED_10_ was calculated for each lesion. Overall, patients with primary NSCLC received higher BED_10_ compared to patients with lung metastases (Fig. 1), with a median BED_10_ of 105.6 Gy for primary NSCLC lesions and 93.6 Gy for metastatic lesions (p = 0.0001).

**Table 2:** SBRT treatment characteristics

	Primary NSCLC	Lung Metastases	p-value
Number of Lesions	48	66	
Prescribed Dose (Gy)			0.0001
Median	48	37.5	
Range	(20 – 60)	(16 – 60)	
BED_10_ (Gy)			0.0005
Median	105.6	93.6	
Range	48 – 180	37.5 – 180	
Dmax (Gy)			
Median	72.7	66.2	0.048
Range	(40.5 – 105)	(21.7 – 94)	
Number of fractions			0.013
Median	4	3	
Range	1–5	1–5

### Overall survival and local control

**Fig. 2:**
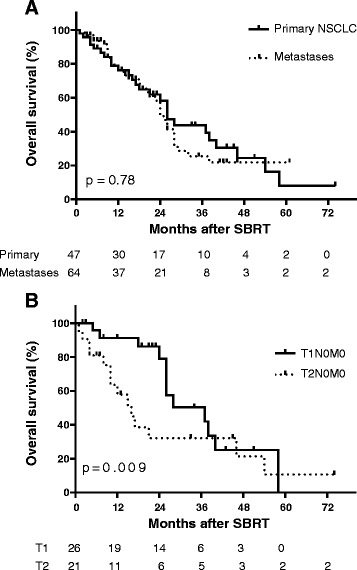
Kaplan-Meier analysis of overall survival for patients with centrally located primary NSCLC and lung metastases (**a**) and for patients with T1N0M0 and T2N0M0 (**b**) lesions. Number of subjects at risk are shown below. Tick marks indicate censored patients

The median follow-up period was 17 months (range, 1–72 months). Median OS for patients with primary NSCLC and lung metastases was 26 and 24, months, respectively (p = 0.78 by log-rank test; Fig. 2a). With respect to only primary NSCLC patients, median OS was 37 months for T1 and 16 months for T2 (p =0.009 by log-rank test; Fig. 2b). One-year OS for T1, T2 and lung metastases was 91.3 % (95 % CI: 69.3 – 97.7), 57.8 % (95 % CI: 32.7 – 75.1) and 77.4 % (95 % CI: 63.4 – 86.6), respectively. Two-year OS for T1, T2 and lung metastases was 79 % (95 % CI: 52.1 – 91.8), 32.1 % (95 % CI: 19.0 – 54.2), and 49.6 % (95 % CI: 33.7 – 63.5), respectively. In univariate analysis of potential prognostic factors for OS, T stage (Wilcoxon-Breslow T1 vs T2 p = 0.0096) and age (continuous log rank p = 0.0105) was significantly associated with OS. However, these variables along with the potential interaction of BED_10_ proved not to be significant in Cox multivariable analysis.

**Fig. 3:**
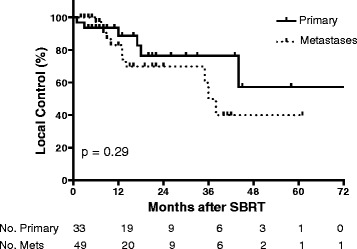
Kaplan-Meier analysis of LC for patients with centrally located primary NSCLC and lung metastases. Number of subjects at risk is shown below. Tick marks indicate censored patients

Lesion response was available for 82 patients and included 33 primary NSCLC lesions and 49 lung metastases. There were 17 local failures; 6 (12.8 %) primary NSCLC and 11 (16.6 %) lung metastases (p = 0.54). The one-year LC rate for primary NSCLC and lung metastases was 88.6 % (95 % CI: 69.4 – 96.2 %) and 82.9 % (95 % CI: 63.4 – 95.7 %), respectively (Fig. 3). The two-year LC rate for primary NSCLC and lung metastases was 76.4 % (95 % CI: 51.5 – 87.0 %) and 69.8 % (95 % CI: 48.0 – 83.8 %), respectively. There was no significant difference for LC between T1 and T2 tumors. In this cohort, prescription dose and BED_10_ did not have a significant effect on LC, with a median BED_10_ of 100 Gy (range 37.5 Gy – 151.2 Gy) for patients with local failures compared to BED_10_ of 100 Gy (range 41.6 – 180 Gy) for those without local failures (p = 0.71). For central primary lung tumors, age, T stage and dose (BED) did not have any significant prognostic impact on local control in this retrospective dataset.

### Doses to organs at risk and toxicities

The maximum point dose (Dmax) was reported for organs at risk and shown as mean Dmax with 95 % CI and median Dmax (Table 3). The mean Dmax to the bronchus was 34 Gy (95 % CI: 24 – 44 Gy), mean Dmax for major vessels was 35 Gy (95 % CI: 31 – 40 Gy), mean Dmax to the trachea was 24 Gy (95 % CI: 18 – 30 Gy), the mean Dmax for esophagus was 14 Gy (95 % CI: 11 – 16 Gy), mean Dmax for heart was 22 Gy (95 % CI: 18 – 25 Gy), mean Dmax to the spinal cord was 10 Gy (8 – 12 Gy) and mean Dmax to skin was 27 Gy (17 – 36 Gy). As a comparison, normal tissue dose constraints recommended by the MD Anderson Cancer Center group for SBRT-treated centrally located lung tumors are shown in Table 3 [11]. The mean and median Dmax values for bronchus, major vessels, trachea, esophagus, heart, spinal cord and skin from patients in the RSSearch® database met or were below the maximum point dose constraint recommended by MD Anderson, although there were a limited number of patients that did have doses to normal structures that exceeded the recommendations.

**Table 3:** Maximum point dose to organs at risk

Organ	Mean Dmax	Median Dmax	MDACC [11]
	(95 % CI)	(range)	Dmax Recommendations (50 Gy/4 Fx)
Esophagus	14 Gy (11 – 16)	11 Gy (0 – 51)	≤ 35 Gy
Bronchus	34 Gy (24 – 44)	38 Gy (26 – 75)	Bronchial tree:
			≤38 Gy
Trachea	24 Gy (18 – 30)	20 Gy (0 – 75)	V35 < 1 cm^3^
Heart	22 Gy (18 – 25)	18 Gy (0.5 – 66)	≤45 Gy
Major vessels	35 Gy (31 – 40)	31 Gy (13–76)	≤56 Gy
Spinal cord	10 Gy (8 – 12)	9 Gy (1 – 24)	<25 Gy
Skin	27 Gy (17 – 36)	17 (9 – 73)	30 < 50 cm^3^

Treatment-related toxicity is shown in Table 4. Fatigue was the most common toxicity and occurred in 5 patients. One patient had an acute Grade 2 cough and one patient had acute Grade 2 pain. One patient had an acute Grade 1 esophagitis. This patient had a 2.9 cm metastatic paratracheal lung metastases treated with 35 Gy in 5 fractions and received a maximum point dose of 40.8 Gy to the esophagus. There were no Grade 3 or higher acute toxicities. The number of late toxicities reported was low. Two patients had late Grade 2 dyspnea, one of these patients also had a late Grade 2 cough. This patient had a 4 cm^3^ primary NSCLC located adjacent to the right hilum. The patient was treated with 48 Gy in 4 fractions and received a maximum point dose of 62.9 Gy to the right lung. There was one patient with a late Grade 2 chest pain and one patient with late Grade 2 pneumonitis. The patient with the Grade 2 pneumonitis had a 66.8 cm^3^ metastatic lesion located in the right peri-hilum region and was treated with 50 Gy in 5 fractions (Dmax 63 Gy). There were no Grade 3 or higher late toxicities.

**Table 4:** Acute and late toxicity following SBRT for centrally located lung tumors

Acute Toxicity	Grade 1	Grade 2	Grade 3	Grade 4/5
Cough	2	1		
Dyspnea	1			
Esophagitis	1			
Fatigue	5			
Nausea	1			
Pain		1		
Late Toxicity	Grade 1	Grade 2	Grade 3	Grade 4/5
Cough	2	1		
Dyspnea		2		
Fatigue	3			
Pain		1		
Pneumonitis	1	1		

## Discussion

In the current observational study which included 111 patients with 114 centrally located early-stage NSCLC or lung metastases, SBRT dose and fractionation regimens varied considerably across centers with doses ranging from 16 – 60 Gy (median 48 Gy) delivered in 1 – 5 fractions (median number of 4 fractions). Overall, patients with primary NSCLC were older and received greater prescribed dose, Dmax and BED_10_ compared to lung metastases. Despite this heterogeneous cohort, we observed OS rates comparable to other published reports of SBRT for the treatment of centrally located lung tumors (Table 5). Overall survival rates range from 12 to 74 %, with patients with advanced disease or lung metastases having worse OS compared to those with Stage I disease [9–17]. Milano *et al.* reported 2-year OS for centrally located lung cancer patients with Stage I, Stage II-III and lung metastases treated with SBRT of 72, 12 and 49 %, respectively [13]. In our study, two-year OS rates for T1, T2 and lung metastases were 79, 32.1 and 49.6 %, respectively, which is in line with published reports.

**Table 5:** Summary of published studies of SBRT-treated centrally located lung tumors

Author	# Patients	Lesion Type	Dose/fraction (Fx)	Median Follow-up (months)	Local Control (years)	Overall Survival (years)
Timmerman [10]	70	T1-T2,N0M0 NSCLC	60-66 Gy/3 Fx	17.5	2-yr: 95 %	2-yr: 54.7 %
Milano [13]	53	Primary, Metastases	30-63 Gy/2.5-5 Fx	10	2-yr: 73 %	2-yr:
						Stage I: 72 %
						Stage II-III:12 %
						Mets: 49 %
Song [9]	32	Stage I NSCLC	40-60 Gy/3-4 Fx	26.5	2-yr: 85.3 %	1-yr: 70.9 %
						2-yr: 38.5 %
Unger [18]	20	Hilar lesions -Primary, Metastases	30-40 Gy/5 Fx	10	1-yr: 63 %	1-yr: 54 %
Haasbeek [12]	63	Stage I-II NSCLC	60 Gy/8 Fx	35	2-yr: 92.6 %	2-yr: 69 %
					5-yr: 92.6 %	5-yr: 49.5 %
Rowe [16]	47	Primary, Metastases	BED_10_: 60–151.2/3-5 Fx	11.3	2-yr: 94 %	NA
					BED ≥ 100 Gy: 100 %	
					BED < 100 Gy: 80 %	
Nuyttens [14]	56	Primary, Metastases	45-60 Gy/5-6 Fx	23	2-yr: 76 %	2-yr: 60 %
					BED > 100: 85 %	3-yr: 53 %
					BED ≤ 100: 60 %	
Chang [11]	100	Primary, isolated recurrences	50 Gy/4 Fx	30.6	3-yr: 96.5 %	3-yr: 70.5 %
			70 Gy/5 Fx			
Bahig [19]	39	T1-T2N0 NSCLC	Median BED_10_: 113 Gy (range 106–180 Gy)	22	2-yr: 89 %	2 yr: 74 %
Schanne [17]	90	Stage I/II	24 – 60 Gy/1-18 Fx	18.8	1-yr: 76 %	1-yr: 72 %
					2-yr: 64 %	3-yr: 29 %
					3-yr: 52 %	
Current study	111	T1-T2 NSCLC, Metastases	20 – 60 Gy/1-5 Fx	17	1-yr: 89 %	2-yr:
					2-yr: 72 %	T1: 79 %
						T2: 32.1 %
						Mets: 49.6 %

For centrally located lung tumors, LC at 2-years after SBRT ranges from 60 – 100 % [10–14, 16, 17]. We observed a 2-year LC rate of 76.4 and 69.8 % for primary NSCLC and lung metastases, respectively, which is comparable to results in the literature for this heterogeneous population. Multiple factors including T stage, tumor size, and BED_10_ have been reported as predictors for LC in other studies of SBRT for central and peripheral lung tumors [4, 13, 22, 23]. A perplexing finding in our study however, is that age, T stage and BED_10_ were not prognostic factors for LC on univariate or multivariate analysis within our cohort. One potential explanation is that there were few patients in our study that received BED_10_ < 100 Gy and which may impact statistical power. Other factors that may contribute include the heterogeneity of the patients and participating centers. Our study included 19 centers with patients treated with SBRT from 2004 to 2014. The experience of the treating centers and technological advancements have most likely improved over time and may contribute to factors that affect statistical considerations.

There are a limited number of multi-center studies that have reported on clinical outcomes for SBRT treatment for central lung lesions and to our knowledge, this is one of the largest to date. Schanne *et al.* recently reported a multi-center study from Germany and Austria, which included 90 patients with centrally located Stage I NSCLC treated with SBRT doses of 24–60 Gy over 1–18 fractions [17]. There was substantial variability in the prescribed dose across centers, which is similar to what we observed in our study. In the German/Austrian study, the median BED_10_ was 72 Gy, and the 1- and 3-year freedom from local progression was 76 and 52 %, respectively. As a comparison, RSSearch® patients with primary NSCLC received a median BED_10_ of 105.2 Gy and 1- and 2-year LC rates were 89.2 and 72.4 %, respectively. The overall higher BED_10_ in our study may be a contributing factor to improved LC compared to results in Schanne *et al.*

Escalating SBRT dose can result in improvements in LC for central lung tumors, however pulmonary toxicities and treatment-related deaths have been reported in patients where high doses per fractions were given and/or critical organs at risk (esophagus, heart, great vessels, brachial plexus) were in the high dose volume region [9–13, 16, 18, 24, 25]. Unger *et al.* reported on 20 patients with hilar lung tumors located next to the mainstem bronchus treated with 30–40 Gy in 5 fractions [18]. In this report, one patient with a large GTV (182 cm^3^) had an acute Grade 2 esophagitis, where the Dmax to the esophagus approached 40 Gy. In our study, we also observed a patient with a para-tracheal metastasis with Grade 1 esophagitis that received Dmax of 40.8 Gy to the esophagus. The MDACC group has recently revised its recommendation of dose constraints for normal tissues and recommend a Dmax of ≤ 35 Gy to the esophagus to limit Grade 2 or higher esophagitis [11]. Patients with central tumors are at higher risk of Grade 3 and higher toxicities compared to patients with peripheral tumors treated with SBRT [26]. The type and severity of toxicities vary for each individual study. Rowe *et al.* reported on 47 patients with central lung lesions treated mainly with 50 Gy in 4 fractions. In this study, toxicity was minimal with four patients (9 %) experiencing Grade 3dyspnea. There were no Grade 4 toxicities, however, one patient with a 5.7 cm pulmonary metastasis abutting the left mainstem bronchus suffered a Grade 5 toxicity and died of respiratory failure [16]. Chang *et al.* reported on 100 patients with primary or recurrent NSCLC treated with SBRT with 13 % of patients with Grade 2 chest-wall pain and 1 % of patients with Grade 3 pneumonitis [11]. There were no Grade 4 or higher toxicities. Overall, reported toxicities in our study were low compared to other published reports, with no Grade 3 or higher toxicities. A limitation of our study, as with retrospective studies, is the potential for underreporting. To address this issue, we did conduct an independent audit of 10 % of patients to verify data completeness and accuracy prior to data analysis, however to completely address the concern of under-reported toxicities, multi-center prospective studies designed to address toxicity are needed.

Another unique feature of this study is that all patients were treated with the CyberKnife® Stereotactic Radiosurgery System with Synchrony® Respiratory Tracking System. The CyberKnife with Synchrony Tracking System uses image-guidance and real-time motion management to accurately deliver high doses of radiation to tumors that move with respiration. Studies reporting SBRT treatment of centrally located tumors using the CyberKnife System have resulted in excellent clinical outcomes with low incidence of toxicities [14, 18, 19]. A potential advantage of the CyberKnife System using Synchrony is the capability to track tumor motion, leading to a reduced margin (typically 3–10 mm) [27–29], in comparison with linac-based systems that use breath-hold techniques or respiratory gating, where the tumor position is generated from different phases of respiration and typically include an 5–10 mm margin to compensate for tumor motion with an addition small margin for set-up uncertainty [13, 30, 31]. This reduction in margin may spare adjacent normal tissues from receiving high doses of radiation, thereby resulting in low toxicities. Future studies need to be done to investigate the long-term clinical outcomes. In principle SBRT treatments maximize the delivery of prescribed dose to the tumor with a rapid fall off of dose to the surrounding normal tissue. A potential advantage of the CyberKnife System is it’s intrinsic ability to create a treatment plan which includes hundreds of non-isocentric beams cumulating the dose in target volume and minimizing the dose fall off, thereby sparing organs at risk. Studies have shown improvements in treatment plan quality when using non-coplanar beams with sufficient quality and quantity [32, 33]. Rossi *et al.* showed that CyberKnife treatment plans generated with non-coplanar beams were superior in regards to sparing organs at risk compared to plans generated with only coplanar beams and that plan quality improved with increasing number of involved beams [34]. It is important to note that treatment plan generation is highly operator dependent and studies assessing clinical outcomes as a result of CyberKnife treatment plan optimization for central lung patients have not been reported. There is also scarcity of literature on the comparative efficacy and toxicity of various SBRT techniques. Furthermore, future studies need to be done to assess the effects of SBRT on long-term outcomes on centrally-located lung tumors.

Patient registries, like RSSearch®, have the ability to accumulate data from a large number of patients in a relatively short time and thus, outcomes from patient registries can be reported years before outcomes of prospective clinical trials. Studies from patient registries will not replace randomized clinical trials, however outcomes from patient registries can complement cooperative group studies as well as generate hypotheses for future studies. In summary, this observational study it is one of the largest patient registries to report treatment management practices of SBRT for centrally located tumors in a real world setting. It adds to the increasing body of evidence for SBRT for centrally located lung tumors and provides support for future prospective studies to define the optimal SBRT dose for centrally located lung tumors.

## Conclusions

In this study, patients with centrally located primary NSCLC and lung metastases were treated with SBRT doses ranging from 16 – 60 Gy (median 48 Gy) and delivered in 1–5 fractions. Overall, patients with primary NSCLC were older and received higher SBRT doses as compared to patients with lung metastases. Despite these differences, we observed excellent LC and OS rates. Toxicities were low, however, low grade toxicities were observed in patients with large tumors or where normal tissues where included in the high dose volume region. This study contributes to the growing evidence to support SBRT for the treatment of centrally located lung tumors, however, prospective studies are needed to define the optimal SBRT dose/regimens while respecting normal tissue dose constraints to minimize toxicities.
